# Diagnosis and Management of a Massive Eight-Centimeter Acquired Tracheoesophageal Fistula

**DOI:** 10.7759/cureus.43689

**Published:** 2023-08-18

**Authors:** Andrew Engel-Rodriguez, Marilee Tiru-Vega, Jesus Merced-Roman, Vanessa Fonseca-Ferrer, Natalie Engel-Rodriguez, Yomayra Otero-Dominguez, William Rodriguez-Cintron

**Affiliations:** 1 Internal Medicine, Veterans Affairs (VA) Caribbean Healthcare Systems, San Juan, PRI; 2 Pulmonology and Critical Care, Veterans Affairs (VA) Caribbean Healthcare Systems, San Juan, PRI; 3 Internal Medicine, San Juan Bautista School of Medicine, Caguas, PRI; 4 Pulmonary and Critical Care Medicine, Veterans Affairs (VA) Caribbean Healthcare Systems, San Juan, PRI

**Keywords:** large tracheoesophageal fistula, surgical repair, how to manage tracheoesophageal fistula, conservative, surgery, management, diagnosis, benign acquired tracheoesophageal fistula, tracheoesophageal fistula

## Abstract

Here, we present the case of a 61-year-old veteran Hispanic male with recurrent aspiration pneumonitis, aerophagia, tympanic abdominal bloating, and a positive Ono's sign; symptoms present were secondary to diagnosed tracheoesophageal fistulas (TEFs). TEFs are abnormal connections between the esophagus and the trachea. In adult cases, several risk factors have been identified for acquired cases, which include infection, trauma, and cancer. Diagnosis of TEF can be challenging and, in most cases, requires high suspicion. Currently, there are no established guidelines for diagnosing and managing TEF. Clinical assessment and various imaging techniques are essential in the diagnostic process. This article will discuss the etiology, clinical presentation, diagnostic approaches, and management options for acquired TEFs.

## Introduction

A tracheoesophageal fistula (TEF) is a connection between the esophagus and the tracheobronchial tract and is most commonly divided into acquired and congenital etiologies [1]. The acquired type of TEF might be caused by either malignancy or a host of nonmalignant entities. In the congenital form of TEF, a defect in development occurs that is usually associated with esophageal atresia. The incidence of congenital TEFs is 0.03% to 0.04%. TEF from malignancy, which accounts for more than 50% of acquired TEFs, is a devastating complication. The primary tumor location is usually the esophagus, but it can also be the lung, trachea, larynx, thyroid, and lymph nodes [1]. Nonmalignant acquired TEFs occur due to extended periods of endotracheal intubation, traumatic incidents, ingestion of corrosive substances, endoscopic/surgical interventions, radiotherapy, and infectious/inflammatory conditions [1]. Tracheal injuries and erosions, occurring in approximately 0.3%-3% of patients undergoing mechanical ventilation, typically stem from factors such as traumatic intubations, aggressive airway suctioning, and ischemic damage to the tracheal wall caused by pressure exerted by the cuff of endotracheal or tracheostomy tubes [2]. It is worth emphasizing that the more accurate incidence rates likely approach the lower end of this range (0.3%) due to changes in clinical protocols over the past three decades.

The incidence span cited in the study by Couraud et al. might not be current or universally applicable due to variations in medical methodologies, patient demographics, and reporting criteria [2]. Symptoms of TEF often manifest three to four weeks after an injury related to the tracheal cuff, making it challenging to diagnose and manage in the early stages [3]. The clinical presentation of TEF can vary depending on its location, size, and rate of development. Burt et al. conducted a study involving over 200 patients, where the primary symptoms and signs observed were as follows: cough (56%), aspiration (37%), fever (25%), dysphagia (19%), pneumonia (5%), hemoptysis (5%), and chest pain (5%) [4]. In non-ventilated patients, uncontrolled coughing after swallowing, also known as "Ono's sign," is a specific indication of TEF [3]. In patients who are sedated and on mechanical ventilation, the presence of a persistent air leak in the ventilator circuit despite proper cuff inflation should raise suspicion of TEF. Additional signs that may be observed include abdominal bloating during ventilation, decreased tidal volume, worsening oxygenation, recurrent pulmonary infections, and repeated unsuccessful attempts to wean from the ventilator. Since TEFs are unlikely to heal independently and can eventually lead to respiratory complications and death, assessing the risk and promptly initiating diagnostic measures is crucial.

## Case presentation

This is a 61-year-old Hispanic man with a medical history of hypertension, type 2 diabetes mellitus, and seronegative rheumatoid arthritis. The patient was transferred to our institution from a trauma-specialized hospital after a prolonged hospitalization following a motor vehicle accident that resulted in multiple injuries, including blunt chest trauma, bilateral hemothorax, multiple pelvic fractures, bilateral rib fractures, and respiratory failure requiring mechanical ventilation. Initial stabilization interventions included bilateral femur external fixation, bilateral thoracostomy, left rib open reduction, and internal fixation. The patient developed multiple complications during the hospital course, including renal failure requiring renal replacement therapy as well as bacterial and fungal bloodstream infections. Due to prolonged respiratory failure and failure to wean from mechanical ventilation, tracheostomy and gastrostomy were performed for respiratory support and nutritional needs.

**Figure 1:**
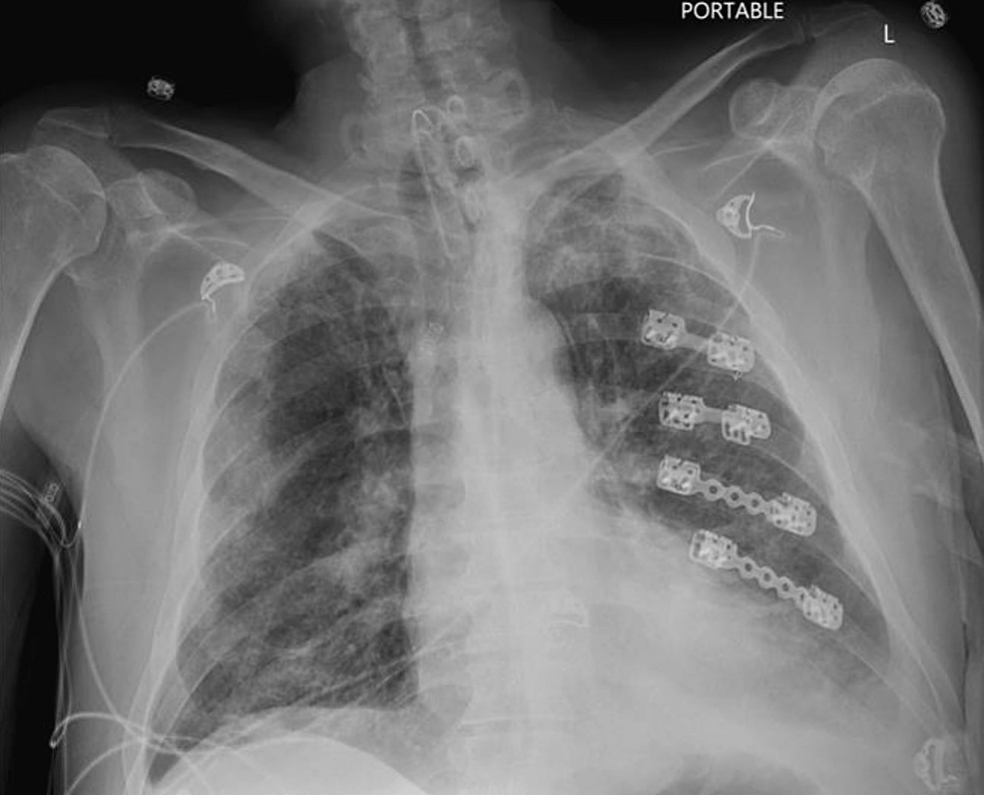
Admission chest x-ray Prominent bilateral interstitial lung marking with partial consolidation of the left lower lobe with effacement of the left hemidiaphragm and retrocardiac region. Multiple right and left rib cage fractures; status post open reduction and internal fixation of the left fifth through left eighth ribs.

Upon arrival at our institution, the patient was oriented in person and place. He received oxygen through a tracheal mask with a 50% fraction of inspired oxygen. The patient presented fever, tachypnea with 30 breaths per minute, a leaking tracheostomy cuff, and scattered abnormal breath sounds upon auscultation. Chest x-ray revealed evidence of bilateral bronchopneumonia (Figure 1).

**Figure 2:**
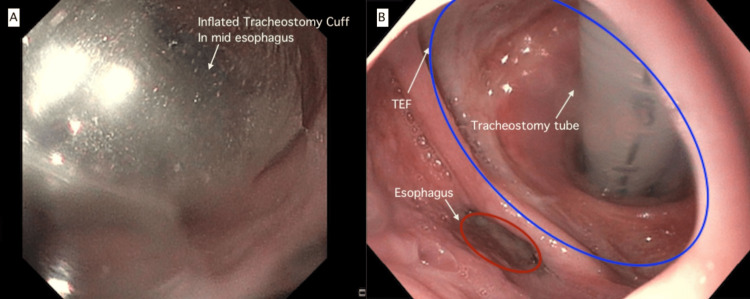
Esophagogastroduodenoscopy (A) Tracheostomy cuff visualized through an opening in the anterior wall of the esophagus, consistent with a TEF. Otherwise normal appearing mucosa of the mid and distal esophagus. (B) The upper esophageal sphincter at 16 cm from incisors, TEF observed just below the UES, extending 2 cm below (down to 18 cm from incisors) with an anterior esophageal wall defect of 8 cm circumferential (blue circle). Esophagus (red circle). TEF: Tracheoesophageal fistula; cm: Centimeters; UES: Upper esophageal sphincter.

Due to increased work of breathing, the patient was placed on mechanical ventilation and empirically started on broad-spectrum antibiotics for suspected hospital-acquired pneumonia/ventilator-associated pneumonia. He was admitted to the intensive care unit (ICU) for further management. The patient experienced progressive aerophagia, vocalization despite tracheostomy, belching, and cough. He became intolerant to enteral feeding and had recurring emesis and aspiration pneumonitis episodes. Physical examination revealed a distended abdomen with audible bowel sounds, a hyper-resonant percussion note, and no tenderness or guarding upon palpation. Abdominal x-ray showed widespread distension of the small and large bowel loops but no signs of obstruction. Clinical suspicion of a TEF arose, but the patient could not undergo conventional imaging tests due to his inability to swallow. A chest computed tomography (CT) scan was performed, which did not support a TEF. Given the persisting clinical suspicion, an endoscopic gastroduodenoscopy (EGD) was conducted by the gastroenterology service. EGD revealed visualization of the tracheostomy cuff through an opening in the anterior wall of the esophagus (Figure 2, Panel A). The tracheostomy cuff was deflated, and the TEF was observed just below the upper esophageal sphincter, extending 2 cm below (18 cm from the incisors), with an eight-centimeter circumferential defect in the anterior esophageal wall (Figure 2, Panel B).

**Figure 3:**
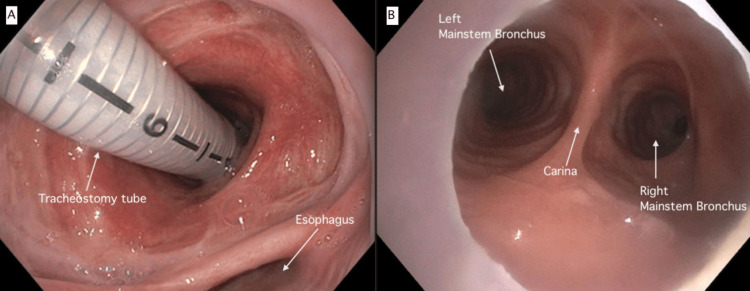
Nasal endoscopy and bronchoscopy through tracheostomy (A) Nasal endoscopy after manipulation of the tracheal tube by ENT where the endotracheal tube was repositioned placing the cuff distal to TEF defect and inside the main airway. (B) Bronchoscopy through the tracheostomy showed that the endotracheal tube was repositioned inside the main airway at approximately 2 cm from the main carina. Trachea had a normal appearance without lesions or edema, and the carina had a normal appearance. ENT: Otorhinolaryngology; TEF: Tracheoesophageal fistula; cm: Centimeters.

Upon identification of the TEF, conservative measures were implemented, including maintaining the head of the bed at an elevation of 45 degrees or higher, administering anti-reflux therapy, frequent oral suctioning, continued treatment for pulmonary infection, and continued supplemental oxygen as needed. Otorhinolaryngology (ENT) and pulmonary services performed nasal endoscopy (Figure 3, Panel A) and bronchoscopy through tracheostomy. To alleviate pressure and avoid further injury to the area, the tracheostomy tube was exchanged from an extended-length tracheostomy to an adjustable neck tracheotomy and was repositioned to have the cuff distal to the tracheoesophageal fistula. The adjustable tracheostomy tip was approximately 2 cm from the main carina (Figure 3, Panel B). Given the extent of the defect, feeding through the gastrostomy resulted in excessive gas and reflux. For such reason, a jejunostomy feeding tube was placed, facilitating enteral feeding at an improved rate. As our community hospitals lacked the surgical expertise to repair such a complex defect, the patient was scheduled to be transferred to a peripheral hospital for TEF repair after nutritional optimization and resolution of all infections. The patient was successfully weaned from mechanical ventilation prior to surgery. Once clinically stable, the patient was transferred, and surgical repair by an interdisciplinary team of surgeons consisting of general surgery, head and neck, and plastic surgery was performed.

## Discussion

TEF diagnosis typically involves clinical assessment, radiographic imaging, and endoscopic examination. Barium swallow, CT scans, bronchoscopy, and endoscopy are commonly employed together as they provide complementary information for diagnosis and treatment [5]. There are no established guidelines for the optimal evaluation and diagnosis of TEFs. The traditional method of diagnosing TEF involves contrast-enhanced esophagography, which reveals the displacement of contrast material into the lungs in up to 70% of the cases [2]. Barium preparation is preferred over Gastrografin due to its high osmolarity, which may lead to pulmonary edema, pneumonia, or even death. In contrast, aspiration of small amounts of barium appears to have limited clinical significance [6]. In mechanically ventilated patients or those unable to swallow, contrast studies may not be feasible, and in such cases, chest CT scans with three-dimensional reconstruction and oral or intravenous contrast medium can serve as an alternative. This imaging technique helps locate the fistula, determine the underlying cause, and examine the anatomy of the trachea and esophagus [7]. Bronchoscopy and/or endoscopy are generally recommended to confirm the findings from imaging studies and precisely locate and measure the defect [5]. However, identifying smaller fistulas can be challenging, particularly when the mucosa is inflamed and swollen. Administration of orally administered methylene blue before bronchoscopy, with observation of bubbles leaking into the airway, has been suggested as a helpful method for identifying small fistulas [8]. Unconventionally, capnography has been employed to diagnose TEF [9]. Capnography measures metabolic and respiratory functions and monitors the atmospheric carbon dioxide (CO_2_) concentration. This provides breath-to-breath analysis and records continuous ventilatory status during procedures. A spike in end-tidal CO_2_ during EGD may facilitate the identification of TEF due to gas passage from the airway to the esophagus [9].

Spontaneous closure of TEF is uncommon, and the prognosis is unfavorable without treatment. Median survival is around three months, and the management of acquired nonmalignant TEF requires a collaborative approach involving thoracic surgery, gastroenterology, and interventional pulmonary experts [3]. Careful evaluation of the cause, size, anatomy (tracheal and esophageal), patient comorbidities, and the risk-benefit ratio of various repair options is crucial when determining the optimal treatment approach. Typically, surgical intervention with the intention of cure is performed for nonmalignant TEFs [10]. Currently, there is limited data and no consensus or guidelines regarding the optimal management of TEF. Clinicians may exhibit significant variation in their practices, but the field evolves as interventional pulmonology expertise grows. Several general measures should be implemented, including discontinuing oral intake, maintaining the head of the bed at an elevation of 45 degrees or higher, providing anti-reflux therapy, frequent oral suctioning, treating pulmonary infections, and administering supplemental oxygen (if necessary) [5]. Nasogastric tubes should be removed if present. In some cases, a gastrostomy tube may be inserted into suction gastric contents, potentially reducing further leakage from gastroesophageal reflux, particularly in mechanically ventilated patients or those with TEF in the lower one-third of the esophagus. Nutritional support can be provided through gastrostomy, jejunostomy, or total parenteral nutrition [5]. Extubation is preferred to minimize shear stress but may not always be feasible. In cases where extubation is not possible, bypassing the site of the TEF can be done by advancing the endotracheal tube (ETT) or using an extra-long tracheostomy tube, ensuring that the cuff is positioned below the fistula and will be inflated with the minimum amount of air that seals the trachea [5]. The formation of a fistula is a gradual process. Inflammation around the area binds the walls of the trachea and esophagus, preventing mediastinitis from occurring [11]. The notion of spontaneous fistula healing is misleading because the edges of the fistula become covered with epithelial cells. In numerous cases, the formation of the fistula is linked to extensive destruction of the trachea caused by the same mechanism of ischemic necrosis. In such cases, treatment typically involves the surgical removal of the affected portion of the trachea and the creation of a direct connection between the remaining ends through terminal-terminal anastomosis [11].

The primary goal of treating benign tracheoesophageal fistula through surgical intervention is curative. However, not all patients or lesions are suitable for surgical repair. Surgical repair of TEF is a technically challenging procedure that involves approaches such as cervicotomy, cervicosternotomy, or thoracotomy. Therefore, esophageal and tracheal surgery expertise is crucial for successful outcomes. The choice of surgical approach depends on the fistula size [1,12]. The preferred surgical approach for definitive treatment involves directly closing the tracheal and esophageal defects. This approach is suitable for patients with small fistulas and a normal trachea. Alternatively, for patients with associated tracheal stenosis or a wide tracheal defect that cannot be repaired directly, tracheal resection and anastomosis are performed along with primary esophageal repair [13]. It is generally recommended to delay surgery until patients have been weaned off mechanical ventilation as positive pressure ventilation has been associated with a higher incidence of complications such as anastomotic dehiscence and restenosis following surgery [1,14]. Similarly, immediate extubation after surgery is desired to reduce the risk of these complications. Successful closure of the fistula has been reported in 75% to 94% of patients who undergo surgical intervention for benign TEF, with median follow-up times ranging from 23 months to 12.5 years [15,16]. Most patients can resume oral intake and do not require prolonged mechanical ventilation after surgery. In a retrospective study, patients who underwent surgical intervention for benign TEF had a lower likelihood of requiring reintervention compared to those who underwent non-surgical interventions such as stenting [17].

Patients with nonmalignant lesions who are not suitable candidates for or whose lesions are not amenable to surgical repair should receive palliative or local therapy [5]. Such interventions are also appropriate for patients requiring a temporary solution before surgery. The preferred approach in nonmalignant TEFs is to use airway and esophageal stents. Single stenting is generally preferred over double stenting to avoid long-term interference with the healing process caused by constant friction and radial pressure between the stent walls [5]. The choice of stent depends on which site can achieve the best fit and sealing effect, considering the available expertise. Double stenting may be considered if a single stent fails to seal the defect adequately [5]. Gastrointestinal over-the-scope-clipping (OTSC) is an emerging technique that enables the closure of gastrointestinal defects, including fistulas. The OTSC device is attached to an applicator integrated into the tip of an endoscope. However, it requires soft and expandable tissue to be effective, limiting its application to the gastrointestinal side of the TEF [18]. Limited case reports suggest satisfactory closure of TEFs using OTSC, but further studies are needed to comprehensively evaluate the outcomes of this technique in this patient population [18,19]. In individual cases, closure of small TEFs has been attempted using local injection of tissue adhesive, fibrin glue, vascular plugs, septal occluders, or silicon rings, yielding variable success rates [5,20].

## Conclusions

Tracheal injuries and erosions are typically caused by traumatic intubations, aggressive airway suctioning, and ischemia of the tracheal wall due to compression by the endotracheal or tracheostomy tube cuff. Prompt diagnosis of TEF can be challenging due to delayed symptomatic presentation. There are no guidelines to direct the diagnosis or management of the pathology. Clinical assessment and imaging techniques like barium swallow, CT scans, bronchoscopy, and endoscopy aid in diagnosis. Management of TEF requires a multidisciplinary approach, and surgical repair is regarded as the primary curative treatment for suitable candidates. A surgeon who undertakes the care of a patient with a TEF must have a thorough understanding of its etiology, diagnosis, and therapy to achieve a successful outcome. However, palliative or local therapies using stents or other interventions may be considered for patients who are not good surgical candidates, require bridging for surgical correction, or have lesions unsuitable for surgery. Further research is needed to evaluate the outcomes and efficacy of emerging techniques. Overall, individualized treatment approaches and close multidisciplinary collaboration among healthcare professionals are essential to optimize the management of TEF and improve patient outcomes.
